# Actions needed to prevent noncommunicable diseases and improve mental health

**DOI:** 10.2471/BLT.18.228700

**Published:** 2019-02-01

**Authors:** Nicholas Banatvala, Svetlana Akselrod, Douglas Webb, Tim Sladden, David Hipgrave, Miriam Schneidman

**Affiliations:** a United Nations Inter-Agency Task Force on the Prevention and Control of Non-communicable Diseases, World Health Organization, avenue Appia 20, 1202 Geneva, Switzerland.; b Department of Noncommunicable Diseases and Mental Health, World Health Organization, Geneva, Switzerland.; c Health and Innovative Financing, HIV, Health and Development Group, United Nations Development Programme, New York, United States of America (USA).; d Sexual and Reproductive Health Branch, United Nations Population Fund, New York, USA.; e Health section, United Nations Children’s Fund, New York, USA.; f Health, Nutrition and Population Global Practice, The World Bank Group, Washington DC, USA.

The 2018 Political Declaration of the Third High-level Meeting on Noncommunicable Diseases notes that progress and investment on noncommunicable diseases have been insufficient to meet the health-related targets of the sustainable development goals.^1^ Countries face many challenges in responding to the rapid rise in noncommunicable diseases and improving mental health as part of the 2030 agenda for sustainable development.^2^ These challenges include insufficient political action on noncommunicable diseases; limited government capacity for policy development, coherence and implementation; insufficient domestic and international finance as well as issues around the impact of economic, market and commercial factors on noncommunicable diseases; and weak health systems, including limited progress on achieving universal health coverage.^3^

The United Nations (UN) Inter-Agency Task Force on the Prevention and Control of Non-communicable Diseases has undertaken joint missions to over 25 countries to support government responses to these challenges.^4^ The missions, in which 17 UN entities have participated, have galvanized political support and influenced policy and practice by engaging with governments, who have primary responsibility for tackling noncommunicable diseases, as well as nongovernmental organizations, the private sector and academia.

These missions have identified priorities for an effective national response on noncommunicable diseases. The first priority is developing national frameworks comprising an investment case; a prioritized and costed national plan with sustainable financing; maximized domestic resources; and coordination and accountability structures. The second priority is achieving greater policy coherence across government to effectively deliver national multisectoral action plans on noncommunicable diseases, including stronger legislation and regulation to reduce levels of risk factors, and to build enabling environments for health-promoting behaviours.^5^ The third priority is increasing effective, pro-health partnerships with civil society and the private sector, giving due regard to managing conflicts of interest. The fourth priority is stronger systems for service delivery, including community-based responses with risk communication strategies and social contracting mechanisms.

While the responsibility for addressing these priorities lies with countries, the UN system has a key role in catalysing these responses. The United Nations Development Programme and the World Health Organization (WHO) are already providing support for developing noncommunicable disease investment cases in 15 countries.^6^ Many others are also requesting support. To respond to this demand, a solid mechanism is needed. The task force has proposed a catalytic fund for governments to use in collaboration with development partners, aligning with recent United Nations Economic and Social Council (ECOSOC) resolutions and the 2018 Political Declaration.

The 2017 ECOSOC resolution urged governments, the private sector and donors to explore financing for noncommunicable disease prevention and control, and to mobilize adequate, predictable and sustained resources for the programmatic work of the task force.^7^ In 2018 ECOSOC further called upon the task force and its members to cooperate with philanthropic foundations, civil society and the private sector to identify additional resources to support Member States.^8^ In the 2018 Political Declaration, heads of State and Government called upon WHO to promote action on noncommunicable diseases. This call included the need to coordinate with other UN agencies, development banks and regional and international organizations; explore new financing, implementation, monitoring and evaluation and accountability mechanisms; and develop national noncommunicable disease investment cases. The WHO Independent High-level Commission also recommended that the international community consider a multidonor fund to catalyse financing for the development of national noncommunicable disease and mental health responses and stronger policy coherence at country level.^9^

The 2015 Addis Ababa Action Agenda was clear that noncommunicable disease responses should be financed from domestic resources.^10^ Seven years after the first noncommunicable diseases Political Declaration,^11^ now is the time to catalyse this response and build sustainable health-promoting policies and programmes to reverse the increasing levels of noncommunicable diseases across the world.
